# LKB1 Destabilizes Microtubules in Myoblasts and Contributes to Myoblast Differentiation

**DOI:** 10.1371/journal.pone.0031583

**Published:** 2012-02-14

**Authors:** Isma Mian, Willythssa Stéphie Pierre-Louis, Neha Dole, Renée M. Gilberti, Kimberly Dodge-Kafka, Jennifer S. Tirnauer

**Affiliations:** 1 Center for Molecular Medicine and University of Connecticut Health Center, Farmington, Connecticut, United States of America; 2 Calhoun Center for Cardiology, University of Connecticut Health Center, Farmington, Connecticut, United States of America; Northwestern University Feinberg School of Medicine, United States of America

## Abstract

**Background:**

Skeletal muscle myoblast differentiation and fusion into multinucleate myotubes is associated with dramatic cytoskeletal changes. We find that microtubules in differentiated myotubes are highly stabilized, but premature microtubule stabilization blocks differentiation. Factors responsible for microtubule destabilization in myoblasts have not been identified.

**Findings:**

We find that a transient decrease in microtubule stabilization early during myoblast differentiation precedes the ultimate microtubule stabilization seen in differentiated myotubes. We report a role for the serine-threonine kinase LKB1 in both microtubule destabilization and myoblast differentiation. LKB1 overexpression reduced microtubule elongation in a Nocodazole washout assay, and LKB1 RNAi increased it, showing LKB1 destabilizes microtubule assembly in myoblasts. LKB1 levels and activity increased during myoblast differentiation, along with activation of the known LKB1 substrates AMP-activated protein kinase (AMPK) and microtubule affinity regulating kinases (MARKs). LKB1 overexpression accelerated differentiation, whereas RNAi impaired it.

**Conclusions:**

Reduced microtubule stability precedes myoblast differentiation and the associated ultimate microtubule stabilization seen in myotubes. LKB1 plays a positive role in microtubule destabilization in myoblasts and in myoblast differentiation. This work suggests a model by which LKB1-induced microtubule destabilization facilitates the cytoskeletal changes required for differentiation. Transient destabilization of microtubules might be a useful strategy for enhancing and/or synchronizing myoblast differentiation.

## Introduction

Muscle fibers form in the developing embryo through the fusion of myoblasts into multinucleate myotubes. In adult tissues, muscle stem cells known as satellite cells line the surface of muscle fibers and provide a source of myoblasts for muscle homeostasis, hypertrophy, and repair of injury [1]. In response to differentiation signals, myoblasts withdraw from the cell cycle, re-organize their cytoskeleton, and ultimately fuse into multinucleate myotubes (reviewed in [2]). Upregulation of the transcription factors MEF2 and MyoD occurs early in the process, and this is followed by expression of myocyte specific proteins such as muscle myosin. This differentiation process has been modeled in vitro using myoblast cell lines, which differentiate upon switching from standard growth media containing fetal calf serum to differentiation media, which contains a lower percentage of adult horse serum, over the course of three to four days [3].

One of the most dramatic changes observed in cultured myoblasts during differentiation occurs in the microtubule cytoskeleton. Microtubule organization completely changes - from a radial array of individual microtubules that emanate from a single central microtubule organizing center (MTOC) in myoblasts - to a dense longitudinal linear array that originates from a diffuse, perinuclear microtubule organizing network and/or non-centrosomal, cytoplasmic sites in myotubes [4], [5], [6], [7], [8]. The mechanisms of this microtubule reorganization and stabilization remain incompletely understood, but it is clear that they play an important role in (and are not merely a byproduct of) differentiation, because both anti-microtubule drugs and loss of microtubule regulatory proteins greatly impair or prevent differentiation [9], [10], [11], [12], [13], [14], [15].

Myotubes contain a population of elongated, stabilized microtubules with reduced turnover. The microtubule binding proteins demonstrated to have positive roles in myoblast differentiation (MAP4, EB1, EB3) all act to stabilize microtubules and promote their elongation [10], [13], [14]. Thus, forced microtubule stabilization might be expected to promote differentiation. However, the converse is true: treatment of myoblasts with the microtubule stabilizing drug Taxol is reported to block differentiation ([16] and our data presented here). Thus, simple microtubule stabilization is likely to be insufficient to produce this stable, reorganized microtubule array.

Liver kinase B1 (LKB1) is a serine-threonine kinase that was originally identified as the product of the tumor suppressor gene mutated in the familial Peutz-Jeghers cancer syndrome (PJS) [17]. Patients who inherit a germline mutation in a single allele of the STK11 gene that encodes LKB1 develop a syndrome of gastrointestinal polyps; malignant tumors of the gastrointestinal tract and other tissues; and skin pigmentation [18], [19]. Somatic mutations of LKB1 have been observed in other tumor types (reviewed in [20], [21], [22]). Germline deletion of the gene encoding LKB1 is lethal during embryogenesis, and mouse models of heterozygous germline LKB1 mutation have been established in which the animals develop tumors of a similar distribution to human PJS [23].

Dramatic muscle phenotypes have not been reported in human PJS patients or in mouse models of germline LKB1 deletion. Together with the finding that LKB1 gene knockout in skeletal muscle did not produce an obvious phenotype in young animals [24], this data gave the impression that LKB1 did not play a major role in muscle development. Subsequent genetic data, however, has shown important roles for LKB1 in muscle. This includes the finding that both skeletal and cardiac muscle phenotypes developed in older LKB1 knockout mice, with decreased voluntary running, type II muscle fiber atrophy, and loss of hind limb muscle function [25]. LKB1 was also shown to affect the differentiation of mouse embryonic fibroblasts (MEFs) into myofibroblasts, contractile cells that express smooth muscle actin and show acto-myosin contractility [26], [27]. Finally, activation of the LKB1 downstream kinase AMPK was impaired in LKB1 skeletal muscle knockouts [24], and running ability was reduced due to diminished muscle function [28]. These data suggest that LKB1 has a role in muscle function and might contribute to muscle development and/or homeostasis.

Mechanisms by which LKB1 could control muscle differentiation include promoting changes in cell polarity or microtubule stability [29], [30], [31], [32], [33], [34], [35]. LKB1 is proposed to be at or near the top of a network for polarity establishment in some systems including epithelial and neuronal cells [36]. LKB1 has been reported to reduce the stability of microtubules, because introduction of LKB1 into LKB1-null mouse embryonic fibroblasts (MEFs) was able to suppress microtubule growth in an assay that measured the elongation of microtubules following washout of the microtubule destabilizing drug Nocodazole [37]. How or whether these roles of LKB1 in cell polarity and microtubule destabilization are linked is not completely clear.

In this study, we asked whether microtubule destabilization plays a role in skeletal myoblast differentiation, and we tested the role of LKB1 in myoblast microtubule elongation and myoblast differentiation. We found that forced microtubule stabilization blocks myoblast differentiation, suggesting that stabilization alone is insufficient to drive the cytoskeletal changes associated with this process. We found that myoblast differentiation is associated with an initial reduction in microtubule stability that precedes the microtubule reorganization and pronounced stabilization seen in myotubes. We further found that LKB1 suppresses microtubule assembly in C2C12 myoblasts, making it a potential candidate to facilitate this reduction in microtubule stability and/or microtubule reorganization. LKB1 RNAi reduced differentiation in C2C12 cells, and LKB1 overexpression enhanced it, supporting a positive role for LKB1 in myoblast differentiation. LKB1 is thus a candidate to promote myoblast differentiation by reducing microtubule stability early in the differentiation process, analogous to factors that reduce microtubule stability early in mitosis to facilitate formation of the mitotic spindle.

## Results

### Microtubule stabilization prevents myoblast differentiation

**Figure 1:**
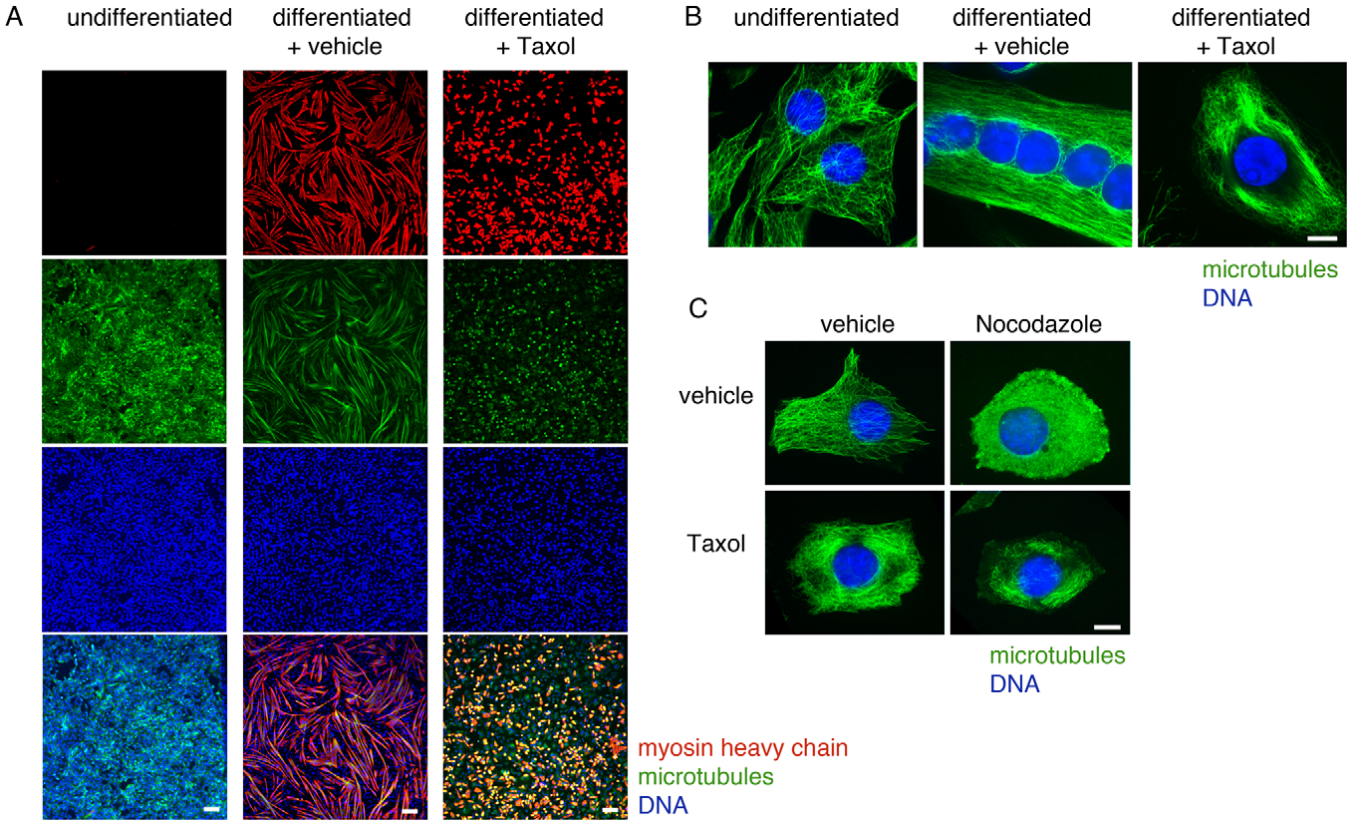
Forced microtubule stabilization prevents differentiation. Differentiation and Taxol treatment both resulted in stable, longitudinally bundled microtubules, but Taxol-treated cells fail to elongate or fuse. C2C12 cells were allowed to proliferate in growth media or cultured in differentiation media for 3 days in the presence of vehicle alone or 100 nM Taxol. Cells were fixed and immunofluorescence for myosin heavy chain (red) and microtubules (green) performed, with imaging at 4× (A) or 100× (B) magnification. Undifferentiated cells lacked myosin heavy chain expression (A) and microtubules were organized in a radial array (B). Differentiated cells treated with vehicle alone became elongated and multinucleate, expressed abundant myosin heavy chain (A), and microtubules were organized in a longitudinally bundled array (B). Cells treated with Taxol during differentiation expressed myosin heavy chain (A) and organized microtubules in longitudinal bundles (B), but they failed to elongate or become multinucleate. C. Taxol treatment stabilizes C2C12 cell microtubules. Cells were treated with vehicle alone or 100 nM Taxol, followed by microtubule depolymerization with 5 µM Nocodazole for 1 hour. Cells were fixed and immunofluorescence for tubulin (green) performed. Control cells showed a radial array of single microtubules, whereas cells treated with Taxol showed microtubules organized in longitudinal bundles. Control cells treated with Nocodazole showed complete microtubule depolymerization, whereas Taxol-treated cells treated with Nocodazole showed resistance to this depolymerization. Bars: A, 100 µm; B and C, 10 µm.

Differentiated myotubes show dramatically stabilized microtubules. If simple microtubule stabilization were responsible for the cell shape changes that precede cell fusion, microtubule stabilizing drugs might be expected to accelerate the process. We tested this by differentiating C2C12 cells in the absence and presence of the microtubule stabilizing drug Taxol. Cells were grown in standard growth media until they reached near-confluence, followed by replacement with media containing 2% horse serum (differentiation media). The differentiation media was supplemented with the dilution vehicle alone or 100 nM Taxol, a potent microtubule-stabilizing drug. In control undifferentiated myoblasts, single microtubules were organized in a radial array (Figure 1A). In control differentiated myotubes, microtubules were arranged in an array of dense, linear bundles consistent with massive stabilization, and they expressed the differentiation marker muscle myosin heavy chain (Figure 1B). The addition of Taxol during differentiation also created a dense microtubule array, but it prevented cell elongation and fusion and caused reduced myosin expression (Figure 1C). Thus, rather than promoting myoblast differentiation, hyperstabilization of microtubules with Taxol completely prevented it.

We next tested whether a shorter period of exposure to microtubule-altering drugs affected myoblast differentiation. We cultured C2C12 cells in differentiation media containing vehicle, 200 nM Taxol, or 200 nM Nocodozole. After two days of this incubation, culture was continued in differentiation media lacking drugs. We found that treatment with both Nocodazole and Taxol prevented cell elongation and fusion, consistent with an important role for microtubules in these processes (Figure S1). Within six hours after drug washout, cells treated with Taxol remained rounded, but cells treated with Nocodazole showed dramatic elongation, similar to controls. By 24 hours, cells treated with Nocodazole differentiated as well as controls, as assessed by morphology in phase contrast images (data not shown). This experiment showed that exposure to Nocodazole followed by drug washout was more conducive to differentiation than was exposure to Taxol followed by drug washout, and could potentially be used to synchronize cells prior to fusion. It also suggested the possibility that microtubule destabilization could contribute to myoblast differentiation.

### Differentiating myoblasts show transient microtubule destabilization

**Figure 2:**
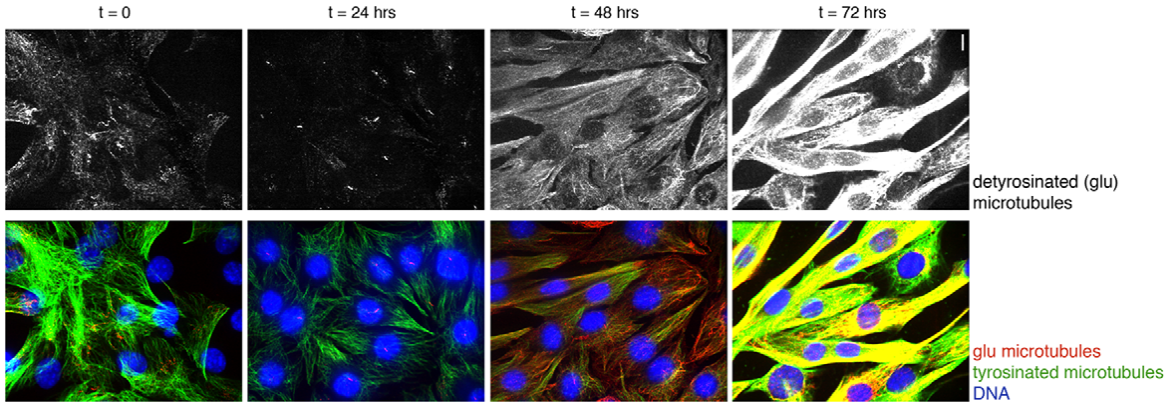
Myoblasts show a transient reduction in stable microtubules prior to an ultimate increase in microtubule stabilization. Cells were differentiated by serum switch for the indicated times, fixed, and immunofluorescence for detyrosinated (glu-) tubulin, a marker of stabilized microtubules, and tyrosinated microtubules, a marker of more dynamic microtubules, was performed. Upper panel shows detyrosinated tubulin, which was reduced at the 24 hour time point and then progressively increased over the next two days. Short linear structures visible with this antibody at 24 hours are primary cilia. Bottom panel shows glu- and tyrosinated tubulin immunofluorescence merged with DNA staining. Bar, 10 µm.

To test the possibility that microtubule destabilization contributes to differentiation, we assayed for changes in microtubule stability using immunofluorescence. The alpha-subunit of tubulin in stabilized microtubules undergoes reversible post-translational removal of its C-terminal tyrosine (detyrosination) to expose glutamate as the terminal residue [38]. Glu-tubulin specific antibodies can be used to recognize microtubules containing detyrosinated tubulin that serve as a marker of microtubule stabilization. We found that undifferentiated myoblasts showed a small proportion of glu-tubulin-containing microtubules, as has been previously reported for L6 myoblasts [8]. Within the first day of differentiation, the abundance of glu-tubulin became reduced, and this reduction was then followed by a dramatic increase in glu-tubulin as cells elongated and fused (Figure 2). This finding is consistent with a role for a transient reduction in microtubule stability prior to the subsequent reorganization of the microtubule array and ultimate microtubule stabilization seen in myotubes.

### LKB1 destabilizes microtubules in C2C12 myoblasts

Several microtubule regulatory proteins are candidates to destabilize microtubules during the myoblast differentiation process. One of these is LKB1, which was reported to prevent microtubule elongation in embryonic fibroblasts following Nocodazole washout [37]. To determine whether LKB1 regulates microtubules in myoblasts, we manipulated LKB1 levels and assayed microtubule elongation in C2C12 cells following Nocodazole washout.

**Figure 3:**
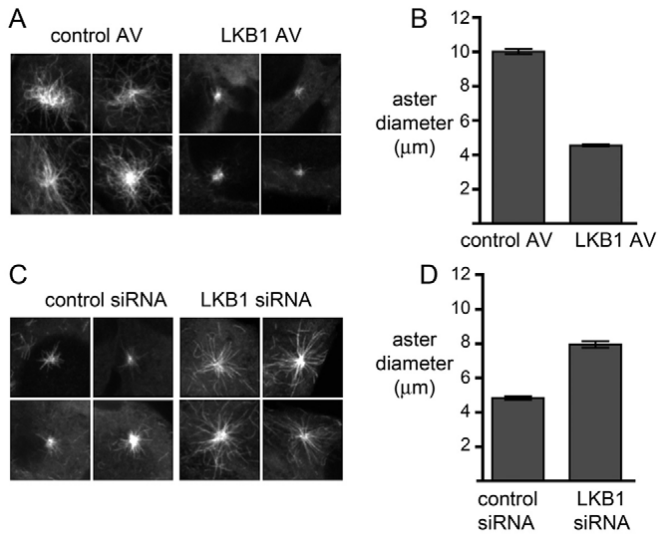
Overexpression of LKB1 suppresses microtubule assembly, and LKB1 RNAi increases it. (A) C2C12 cells were infected with a control adenovirus (control AV) or an adenovirus that drives expression of wild type human LKB1 (LKB1 AV) for 24 hours. Cells were treated with Nocodazole for 1 hour followed by a 7 minute washout, fixation, and microtubule immunofluorescence. Representative microtubule asters are shown. (B) Quantification of aster diameter for A, expressed as mean diameter+/−s.e.m. (C) C2C12 were cells transfected with a control siRNA or an siRNA that targets expression of LKB1 and treated with Nocodazole for 1 hour followed by a 2 minute washout, fixation, and microtubule immunofluorescence. Representative asters are shown. (D) Quantification of aster diameter for C. At least two experiments were done for each condition. See Fig. 4 for LKB1 levels.

We increased LKB1 levels in C2C12 cells using adenoviral overexpression. Cells were infected with an adenovirus encoding LKB1 or a control virus encoding green fluorescent protein (GFP). We first imaged microtubules in asynchronously growing cells to determine whether LKB1 overexpression altered the microtubule array, and we found no appreciable difference in the appearance of microtubules in cells overexpressing LKB1 compared to controls, consistent with a previous report in non-muscle cells (data not shown) [37]. We then performed Nocodazole washout experiments to determine whether LKB1 could regulate microtubule re-growth, a more sensitive assay of microtubule stabilization. Microtubules in cells treated with the control or LKB1 virus were depolymerized with Nocodazole for one hour, followed by drug washout for a brief interval and rapid fixation in methanol. The size of the resulting microtubule asters was measured using tubulin immunofluorescence. This showed that asters in cells treated with a control virus had a mean diameter of 10.0 µm 7 minutes after Nocodazole washout, while cells treated with the LKB1 virus had a mean aster diameter of 4.6 µm, a 54 percent reduction (Figure 3). Thus, excess LKB1 reduced microtubule elongation.

We did the converse experiment of measuring microtubule re-growth in cells with reduced LKB1 levels, using RNA interference (RNAi). Cells transfected with an siRNA targeting LKB1 did not show any obvious differences in the microtubule array compared to cells transfected with a nonspecific siRNA (data not shown). However, following Nocodazole treatment and washout, microtubule elongation was greater in cells with LKB1 RNAi compared to controls. Cells treated with a control siRNA had a mean aster diameter of 4.8 µm 2 minutes after Nocodazole washout, whereas cells treated with LKB1 RNAi had a mean aster size of 8.0 µm, a 67 percent increase. This result of greater microtubule elongation in cells with reduced LKB1 is also consistent with a role for LKB1 in suppressing microtubule elongation in myoblasts.

### LKB1 translocates to the cytoplasm and phosphorylates downstream kinases during myoblast differentiation

**Figure 4:**
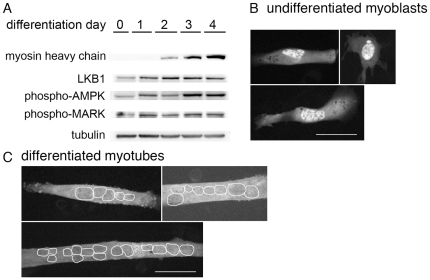
LKB1 levels and substrate activation increases, and LKB1 redistributes to the cytoplasm during differentiation. (A) Western blotting was done on samples from the indicated days of differentiation. Myosin heavy chain (myosin) expression increases progressively. LKB1 levels increase by day 1 and peak at day 2 of differentiation. Levels of the phosphorylated forms of LKB1 substrates AMPK and MARK increase by day 1. Tubulin is shown as a loading control. (B, C) Cells were transfected with GFP-LKB1 as described in text. Representative images from undifferentiated cells (B) and cells cultured in differentiation media for three days (C) are shown. The mean ratio of nuclear to cytoplasmic fluorescence was 3.0 in undifferentiated cells and 1.2 in differentiated cells. Bars, 50 µm.

We next investigated the regulation of LKB1 in myoblast differentiation. We first tested whether LKB1 levels changed during differentiation, by Western blotting for LKB1 at serial time points of a differentiation time course. We confirmed differentiation using phase contrast imaging, as well as Western blotting and immunofluorescence for muscle myosin heavy chain. Western blotting for LKB1 showed that LKB1 levels increased by 56% within the first day of differentiation and remained elevated throughout the differentiation time course (Figure 4A). This early increase in a factor that reduces microtubule elongation further supports a role for reduced microtubule stability prior to eventual microtubule stabilization that occurs in the differentiation process.

Mechanisms of LKB1 activation are not completely understood; however, activation is known to be associated with translocation from the nucleus to the cytoplasm, where LKB1 can phosphorylate target proteins [39], [40]. Thus, we also assayed LKB1 localization during the differentiation process. We were unable to assess LKB1 localization by indirect immunofluorescence using commercially available anti-LKB1 antibodies, so we transfected C2C12 cells with a plasmid encoding GFP-LKB1 [41]. Imaging of GFP fluorescence showed that undifferentiated myoblasts had abundant GFP-LKB1 in the nucleus, consistent with reduced LKB1 kinase activity in the cytoplasm (Fig. 4B). To image GFP fluorescence in multinucleate myotubes, we took advantage of our own observation that cells treated with Nocodazole in differentiation media could be differentiated rapidly by washing out the Nocodazole. This allowed us to circumvent the brief window of GFP-LKB1 expression and the difficulty of transfecting multinucleate myotubes. We treated cells with Nocodazole in differentiation media for two days, transfected them with the plasmid encoding GFP-LKB1, washed out the Nocodazole to allow cell fusion, and imaged GFP-LKB1 the next day. In contrast to undifferentiated myoblasts, multinucleate myotubes expressing GFP-LKB1 showed a greater proportion of GFP fluorescence in the cytoplasm, such that nuclear and cytoplasmic abundance of GFP-LKB1 were equivalent (Fig. 4C). We quantified this by measuring the ratio of nuclear to cytoplasmic GFP fluorescence. In undifferentiated cells, this ratio was 3.0+/−1.5 (n = 24), as opposed to 1.2+/−0.3 (n = 19) in differentiated cells. This is consistent with a shift of LKB1 to the cytoplasmic compartment and an increase in cytoplasmic LKB1 activity during differentiation.

LKB1 phosphorylates several substrates of the AMP-activated protein kinase (AMPK) family, including AMPK and microtubule affinity regulating kinases (MARKs), among others [21], [33]. To test whether the increase in LKB1 levels and cytoplasmic translocation was associated with biologically relevant evidence of LKB1 activation, we assayed for phosphorylation of these substrates during differentiation using Western blotting with phospho-specific antibodies. This revealed a dramatic increase in the phosphorylation of both AMPK (at threonine 172, a known site of activating phosphorylation) and MARK kinases, by day one of differentiation (Figure 4A). On day 1 of differentiation, AMPK phosphorylation increased to 2.3 times and MARK phosphorylation increased to 1.3 times their levels at day 0. Thus, these LKB1 substrates become activated early in differentiation, in parallel with increases in LKB1 abundance and cytoplasmic translocation. Taken together, these findings support the conclusion that myoblast differentiation is associated with LKB1 activation, and associated activation of downstream kinases AMPK and MARKs.

### LKB1 enhances myoblast differentiation

**Figure 5:**
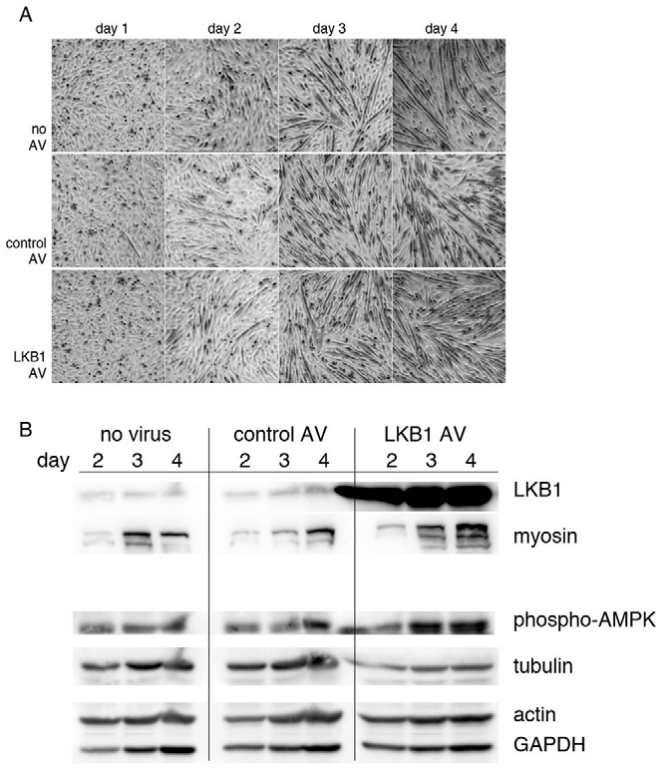
LKB1 overexpression accelerates differentiation. (A) Phase contrast pictures of C2C12 cells uninfected (no virus), infected with an adenovirus overexpressing CRE recombinase and GFP (control AV), or infected with an adenovirus expressing human LKB1, and grown in differentiation media for the indicated number of days. Cells with LKB1 overexpression showed enhanced differentiation. (B) Western blotting shows increased myosin upon LKB1 overexpression. Samples from the indicated days were probed for LKB1, myosin heavy chain (myosin), and phosphorylated AMPK; tubulin, actin, and GAPDH were probed as loading controls.

We next tested whether LKB1 activation plays a positive role in myoblast differentiation. We used LKB1 overexpression and RNAi as described above, followed by differentiation using the serum switch assay. For overexpression, C2C12 cells were infected with the control or LKB1 adenovirus and grown in differentiation media, and differentiation was assayed by phase contrast microscopy and Western blotting for myosin expression. This showed that LKB1 overexpression (16 fold over endogenous levels) caused an increase in differentiation as compared to control virus treatment, with an average of 1.8 fold increased myosin heavy chain expression in cells overexpressing LKB1 as compared to controls (Figure 5). Phosphorylation of AMPK at threonine 172 was increased in cells overexpressing LKB1 at 1.5 times control. Interestingly, the amount of tubulin in cells overexpressing LKB1 was reduced to 53% of control virus-infected cells on Day 1 of differentiation. Thus, LKB1 positively controls differentiation, with supraphysiologic LKB1 levels enhancing the degree of differentiation over cells with endogenous LKB1 levels.

**Figure 6:**
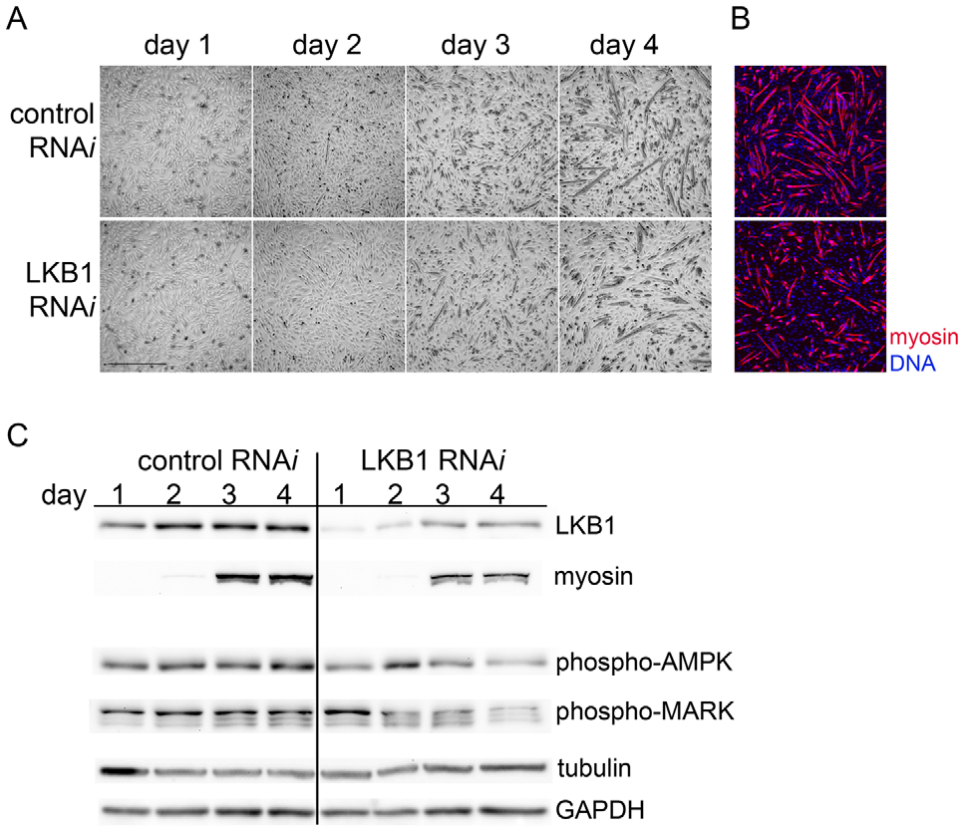
LKB1 RNAi reduces differentiation. (A) Phase contrast pictures of C2C12 cells transfected an siRNA directed against mouse LKB1 (LKB1 RNAi) and controls were and grown in differentiation media for the indicated number of days. (B) Immunofluorescence for myosin and DNA staining done at day 3 of differentiation show reduced myosin expression in cells with LKB1 RNAi as compared to controls. (C) Western blotting shows reduced myosin expression upon LKB1 RNAi, as well as reduced phosphorylated AMPK (phosho-AMPK) and phosphorylated MARK (phospho-MARK). Tubulin and GAPDH were probed as loading controls.

We next tested whether reduced LKB1 levels impair differentiation. We used an siRNA that reduced LKB1 levels to 86% of control on day 1 (Figure 6). During differentiation, LKB1 levels in both control and LKB1 siRNA cells rose over the 4 days of the assay, so that by day 4 of differentiation, LKB1 level in the RNAi sample was 39% of control; this increase probably represented a combination of increased expression due to differentiation and diminution of the siRNA effect. Phase contrast microscopy, immunofluorescence for myosin, and Western blotting for myosin were used to assay morphological and biochemical features of differentiation, and these showed that cells with reduced LKB1 had reduced differentiation (Fig. 6). By Western blotting, the myosin heavy chain level in the LKB1 RNAi sample was 59 and 64 percent of control on days 3 and 4, respectively. LKB1 RNAi caused a reduction in the activation of AMPK, as detected by blotting with phospho-threonine 172 antibodies (43 percent of control at day 4). Unlike controls, cells with LKB1 RNAi showed increased tubulin levels, such that lysates on day 4 of LKB1 RNAi had 1.6 times as much tubulin as on day 1. These assays showed that LKB1 RNAi reduced myoblast differentiation and further support a positive role for LKB1 in the differentiation process that correlates with its ability to phosphorylate substrates.

## Discussion

### A model for microtubule changes during myoblast differentiation

**Figure 7:**
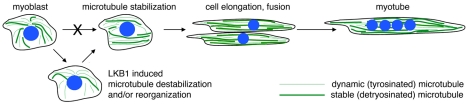
Model. Myoblasts contain a radial microtubule array that is a mixture of dynamic microtubules (identified in vivo by the presence of a C-terminal tyrosine on alpha-tubulin; depicted here as thin green lines) and stabilized microtubules (identified by post-translational detyrosination; depicted here as thick green lines). Fully differentiated myotubes show a linear microtubule array consisting of abundant detyrosinated/stable microtubules. Our data shows that simple microtubule stabilization blocks the formation of myotubes, and a transient decrease in microtubule stabilization precedes cell elongation and fusion into myotubes. We propose a model in which transient microtubule destabilization facilitates microtubule reorganization, and this is then followed by microtubule stabilization. We suggest that LKB1 plays a role in this microtubule destabilization and/or reorganization, which accounts for its role in the differentiation process.

Our results show that microtubule stabilization with Taxol prevents myoblast differentiation, whereas microtubule destabilization with Nocodazole followed by drug washout promotes rapid differentiation with cell fusion. These findings support the conclusion that simple microtubule stabilization, which is seen in fully differentiated myotubes, is alone insufficient to positively impact myoblast differentiation. Based on our findings that (1) microtubule depolymerization followed by re-growth can lead to rapid differentiation, (2) microtubule detyrosination, a marker of stabilization, transiently decreases before it increases, and (3) LKB1, which reduces microtubule elongation, promotes C2C12 differentiation; we favor a model in which early microtubule destabilization might be an important step in a differentiation process that ultimately culminates in marked microtubule stabilization (Figure 7). This might be similar to the initial destabilization of microtubules that is essential to their reorganization and subsequent stabilization during formation of mitotic and meiotic spindles [42], [43]. Similar to mitosis, it is likely that an array of microtubule regulatory proteins contribute to the reduction and subsequent increase in microtubule stability that occur during myoblast differentiation [44]. Live cell imaging experiments with finer temporal resolution will allow us to test the timing and mechanism of a transient reduction in microtubule stability that we believe precedes eventual microtubule reorganization and stabilization seen in myotubes.

### LKB1 positively affects myoblast differentiation

Our experiments show that LKB1, which destabilizes microtubule elongation from centrosomes, is a positive mediator of differentiation, because LKB1 RNAi reduces differentiation, and LKB1 overexpression enhanced it. While several microtubule stabilizing factors have been shown to contribute to myoblast differentiation, we believe LKB1 is the first destabilizing factor demonstrated to contribute to the differentiation process [10], [13], [14]. This finding that a microtubule destabilizing factor promotes myoblast differentiation is not at odds with studies that show critical roles for microtubule stabilizing proteins in differentiation. Rather, we believe that LKB1-induced microtubule destabilization could precede microtubule stabilization effected by EB1, EB3, and MAP4, and that a combination of stabilizing and destabilizing factors is likely to be needed to fine-tune the changes in microtubule organization and membrane events in differentiating myoblast elongation and fusion.

### Mechanism of LKB1 effects on myotube formation

Based on the known downstream functions of LKB1 and the known changes required for myotube formation, there are several mechanisms by which LKB1 could facilitate the differentiation process. Our study establishes microtubule destabilization and reduced cell fusion by LKB1, although it does not rule out other contributions of LKB1 to the process. LKB1 is likely to destabilize microtubules indirectly, as it does not bind to myoblast microtubules in vitro (data not shown). Its role is likely to be associated with LKB1 kinase activity, because LKB1 substrate phosphorylation happens early (by the first day) of differentiation.

There are several LKB1 substrates that could mediate its role in differentiation, and our work does not determine which substrate(s) are involved. Potential mediators include several members of the AMPK family, including AMPK itself and MARKs. Several AMPK substrates are known to regulate microtubule polymerization dynamics, including the microtubule+tip protein CLIP-170 [45] and the microtubule stabilizing protein Tau [46], [47]. However, in contrast to our model, in which the ultimate effect of LKB1 activation was to destabilize microtubules, AMPK was shown to increase the rate of microtubule polymerization, an effect that would be expected to stabilize microtubules, in Vero cells [45]. Additional LKB1 substrates must also be tested. LKB1 could also reduce microtubule stability by suppressing tubulin expression, as lysates from cells with excess LKB1 contained less tubulin than control lysates, and lysates from cells with LKB1 RNAi showed progressive increases in tubulin levels. Further analysis of this possibility is also needed.

Successful myoblast polarization has been proposed to be a prerequisite for fusion competence [14]. LKB1 has roles in both cell polarization and microtubule destabilization, and the degree to which these roles are separable is not completely clear. Thus, LKB1 could be required for myoblast fusion through its effects on microtubules, cell polarization, or both, and its regulation of the two could bifurcate at many regulation points, or could be coupled. While our study does not address this level of mechanistic detail, it does suggest a useful model system in which these questions could be tested.

### Implications for human muscle diseases

Muscle differentiation is not just important during embryogenesis. Muscle undergoes continuous regeneration, through the differentiation of satellite cells to myoblasts, and subsequent myoblast differentiation into myotubes and muscle fibers [48]. When muscles are injured, satellite cells are mobilized for repair by activating new fiber formation. Thus, insights into muscle differentiation might prove useful for enhancing muscle homeostasis and repair in adults. Development of small molecules to manipulate LKB1 activity would be helpful for further investigating and altering the temporal control of differentiation. Another implication of our study is that transient use of microtubule destabilizing drugs might be useful for synchronizing muscle differentiation in vitro or in vivo.

LKB1 has been shown to play a role in the differentiation of neurites [30]. Now with our study showing a role in the differentiation of myoblasts, it would be interesting to see whether LKB1 could promote differentiation of other cell types as well. In a mouse model of the PJS cancer syndrome, LKB1 deletion in myofibroblasts appears to be sufficient to cause a polyposis syndrome [49]. This raises the possibility that introduction of myofibroblasts with wild-type LKB1 expression might play a role in preventing tumorigenesis in these patients.

## Materials and Methods

### Reagents and antibodies

Taxol and Nocodazole were purchased from Sigma Aldrich (St. Louis, MO, USA) and resuspended as 10 mM stock solutions in DMSO. All other chemicals were from Sigma unless otherwise noted.

Antibodies for Western blotting included anti-LKB1 (clone D60C5, Cell Signaling Technology, Danvers, MA, USA) anti-Myosin heavy chain (Developmental Studies Hybridoma Bank; under the auspices of the NICHD and maintained by The University of Iowa, Department of Biology; Iowa City, Iowa, USA), anti-AMPK-alpha (Santa Cruz Biotechnology, Santa Cruz, CA, USA), anti-phospho-AMPK (Clone 40H9, which recognizes phospho-threonine 172, Cell Signaling), anti-MARK (Abcam, Cambridge, MA, USA), anti-phospho-MARK activation loop (Cell Signaling), anti-tubulin (clone DM1A, Sigma), anti-beta-actin (Sigma), and anti-GAPDH (Santa Cruz).

Antibodies for immunofluorescence included FITC-conjugated anti-tubulin (DM1A, Sigma) and anti-myosin heavy chain (Developmental Studies Hybridoma bank), anti-tyrosinated tubulin (clone YL1/2, Millipore, Temecula, CA), and anti-detyrosinated (glu) tubulin (Millipore). Species-specific secondary antibodies conjugated to Alexa-488 and -568 fluors were used (Invitrogen, Carlsbad, CA, USA).

### Cell culture

The mouse myoblast cell line C2C12 was purchased from ATCC (Manassas, VA, USA) and grown in DMEM with 10% fetal calf serum (FCS, not heat inactivated; Atlanta Biologicals, Lawrenceville, GA, USA). Cells were not allowed to exceed 70% confluence during passaging and were discarded after 20 passages (40 days), to minimize depletion of myoblasts from the culture.

Differentiation was induced when cells reached near-confluence using a standard media switch assay. Cells were washed three times with PBS and grown in DMEM with 2% horse serum (Invitrogen) instead of FCS. This induced differentiation within 3–4 days, depending on confluence at the time of the switch and the passage number. These were both matched between controls and other manipulations in all experiments. For immunofluorescence experiments, cells were plated on glass coverslips and allowed to adhere in growth media before differentiation was induced.

### Nocodazole washout and microtubule elongation assay

Cells grown on glass coverslips were incubated in growth media with 10 µM Nocodazole for 1 hour, washed with pre-warmed PBS and pre-warmed media, and incubated in pre-warmed media without Nocodazole for the indicated times (from first PBS wash to ice-methanol fixation). They were fixed by immersion in ice-cold methanol and processed for immunofluorescence as described below. The washout time was counted from the first PBS wash to fixation. Controls from different experiments are not comparable because of pre-warming of PBS and media in some experiments.

Microtubules formed upon washout of Nocodazole were imaged by tubulin immunofluorescence. Approximately 50 randomly chosen 20× fields were imaged, for a total of ∼350–400 cells per condition for LKB1 overexpression and ∼125 cells per condition for LKB1 RNAi. Asters formed from the elongation of microtubules outward in all directions from the cell's centrosome. The diameter of each aster was measured by drawing a line across the aster at its maximum diameter in a single focal plane using the region measurements tool in MetaMorph software. Because the asters were small and the cells flat, a single focal plane contained the entire length of the microtubules. Values are expressed as mean+/−standard error of the mean.

### Transfections for LKB1 RNAi and GFP-LKB1 expression

LKB1 RNAi was done with an siRNA oligo that targets the mouse LKB1 coding sequence with the sequence 5′-GGGUACUUCCGCCAGCUGAtt-3′ (Sigma). Cells were electroporated with the siRNA using a nucleofector (Lonza USA, Walkersville, MD, USA) according to the manufacturer's protocol, in Solution V with program T-017. Approximately 2×10^6^ cells were transfected per reaction with 7–8 µg of siRNA. Controls were treated identically with a control siRNA (Sigma) or omission of the siRNA.

GFP-LKB1 encoded in a plasmid was purchased from Addgene (Cambridge, MA, USA). Transfection of undifferentiated cells was done by nucleofection as above with 3 µg of DNA. For differentiated cells, cells were grown in differentiation media in the presence of 200 nM Nocodazole to prevent cell fusion, transfected with 3 µg of DNA, and plated in differentiation media without Nocodazole for one day prior to imaging, at which point cells had fused as well as controls that were differentiated for the same time period in the absence of Nocodazole.

### Adenoviral LKB1 overexpression

LKB1 overexpression was done by adenoviral infection according to an institutional biosafety committee-approved protocol with a virus encoding human LKB1 (Ad-STK11, Vector Biolabs, Philadelphia, PA, USA). Control cells were infected with a control virus (CRE-GFP, Vector Biolabs). Cells were infected at a multiplicity of infection (MOI) of ∼5 viral particles per cell in differentiation media at the start of the experiment. Differentiation media was changed as needed without re-addition of virus.

### Immunofluorescence, microscopy, and analysis

For phase contrast pictures, cells were imaged in tissue culture plates using an inverted microscope (Nikon Instruments, Melville, NY, USA) at 4× magnification. Random regions of the well were imaged to avoid bias.

For immunofluorescence, cells were grown on glass coverslips and fixed in ice-cold methanol for 5 minutes or in freshly made 4% formaldehyde at room temperature for 20 minutes. Cells were permeabilized in Tris-buffered saline (TBS) with 0.1% Triton X100 and blocked in 2% bovine serum albumin (BSA, Fisher Scientific) in TBS/0.1% Triton X100 for 1 hour at room temperature. Primary and secondary antibodies were diluted in block solution and incubated for 1 hour at room temperature. DNA was stained with Hoechst 33342 (Sigma) at 10 µg/ml for 2 minutes. Coverslips were mounted in media containing 0.5% 0-phenylenediamine in 20 mM Tris pH 8.8 and 90% glycerol and sealed with nail polish. Images were acquired at 4×, 60×, or 100× magnification with a Yokogawa CSU-10 spinning disk confocal head (Perkin Elmer, Wellesley, MA) onto an ORCA AG CCD camera (Hamamatsu Photonics, Bridgewater, NJ). Image acquisition, processing, and analysis were done using MetaMorph software (Molecular Devices Corp, Sunnyvale, CA). All low-power fields were selected randomly to avoid bias.

GFP-LKB1 expression in nucleus versus cytoplasm was calculated by measuring the average pixel intensity in a background-subtracted 20×20 pixel square placed over the nucleus or cytoplasm and taking a ratio for each individual cell, using the region measurements tool in MetaMorph software.

### Western blotting

Cells were washed in PBS and either scraped into lysis buffer (150 mM NaCl, 50 mM Hepes pH 7.4, 2 mM EGTA, 2 mM MgCl_2_, 0.1% Triton X100, with protease inhibitors PMSF, NaF, NaVO_4_, pepstatin, chymostatin, and leupeptin) or trypsinized, pelleted, and stored at −80°C, followed by lysis in this buffer for 30 min on ice. (Of note, trypsinization activated phosphorylation of AMPK on threonine 172, the same epitope phosphorylated by LKB1, presumably through calcium-calmodulin dependent protein kinase kinase (CAMKK). Overexpression of LKB1 and growth in differentiation media further increased this phosphorylation over and above that seen with trypsinization). Lysates were cleared by centrifugation at 14,000 G, and protein was assayed with Bradford reagent (Biorad, Hercules, CA, USA). Adenovirus-infected cells were lysed by washing with PBS and scraping directly into sample buffer, followed by bath sonication to shear DNA and loading of equal lysate volume.

Samples were separated on 10% SDS-PAGE gels, transferred to PVDF membranes, and blocked in 2% BSA in Tris buffered saline (TBS) with 0.1% Tween and 0.1% Sodium Azide. Primary antibodies were incubated for 1–2 hours at room temperature or overnight at 4°C, and secondary antibodies were incubated for 1 hour at room temperature. The Enhanced Chemiluminescence Fempto reagent (Thermo Scientific, Rockford, IL, USA) was used to develop blots. Signal was imaged digitally on either a Genesnap (Syngene USA, Frederick, MD, USA) or Biorad (Biorad) system within the gray range of the camera. Blots were multiply probed for proteins of different masses without stripping.

## Supporting Information

Figure S1 **Microtubule destabilization is more conducive to differentiation than is microtubule stabilization.** C2C12 cells were treated with vehicle (control), 200 µm Taxol, or 200 µm Nocodozole and cultured differentiation media for two days, followed by washout and continued differentiation. (A) Phase contrast images at differentiation day 2 (in the presence of drugs) and day 3 (one day after drug washout) show that both Taxol and Nocodazole cause cell rounding and prevent myoblast fusion, but washout of Nocodazole is associated with more substantial cell elongation than washout of Taxol. (B) Corresponding immunofluorescence images were done on cells fixed 6 hours following drug washout. Insets show higher magnification images of multinucleate cells in controls and Nocodazole treated cultures, and cells with single nuclei in Taxol treated cells. This shows that cells treated with both drugs express myosin heavy chain (MHC, red), but only cells treated with Nocodazole show substantial cell fusion, even at 6 hours following washout. Bar, 50 µm. (C) Fusion index from the same time point as shown in B. Ten random 20× fields were imaged for myosin heavy chain and DNA, and number of cells with one nucleus or more than one nucleus was counted. This showed that 60 percent of control cells expressing myosin heavy chain had fused, while only 24 percent of Taxol treated and 40 percent of Nocodazole treated cells had fused.
(TIF)
