# Supplementary figures and images for: LKB1 Destabilizes Microtubules in Myoblasts and Contributes to Myoblast Differentiation
Source: PLoS One. 2012 Feb 14;7(2):e31583. doi: 10.1371/journal.pone.0031583 (PMC3279410; doi:10.1371/journal.pone.0031583)

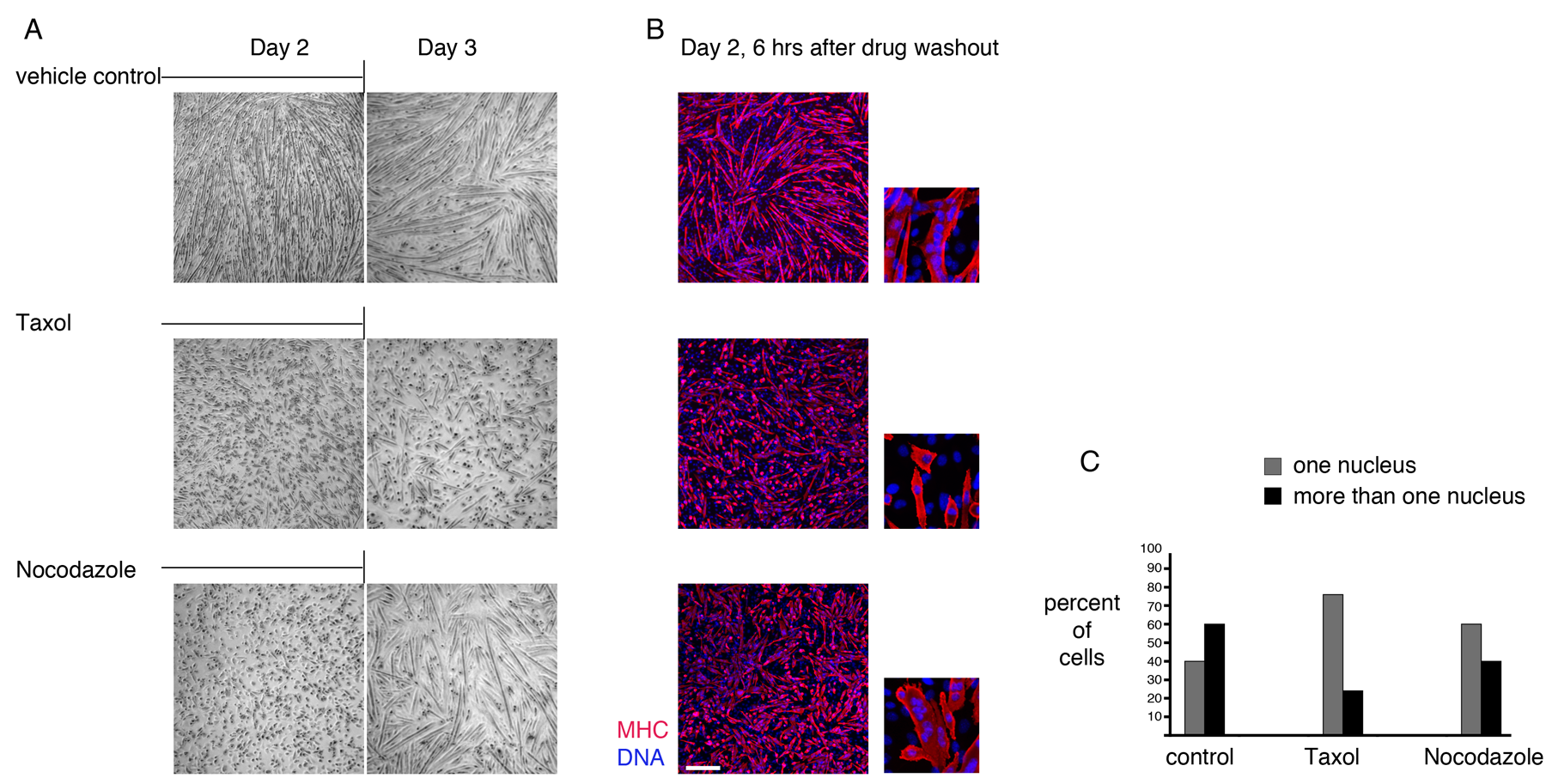

Supplement: Figure S1 — Microtubule destabilization is more conducive to differentiation than is microtubule stabilization. C2C12 cells were treated with vehicle (control), 200 µm Taxol, or 200 µm Nocodozole and cultured differentiation media for two days, followed by washout and continued differentiation. (A) Phase contrast images at differentiation day 2 (in the presence of drugs) and day 3 (one day after drug washout) show that both Taxol and Nocodazole cause cell rounding and prevent myoblast fusion, but washout of Nocodazole is associated with more substantial cell elongation than washout of Taxol. (B) Corresponding immunofluorescence images were done on cells fixed 6 hours following drug washout. Insets show higher magnification images of multinucleate cells in controls and Nocodazole treated cultures, and cells with single nuclei in Taxol treated cells. This shows that cells treated with both drugs express myosin heavy chain (MHC, red), but only cells treated with Nocodazole show substantial cell fusion, even at 6 hours following washout. Bar, 50 µm. (C) Fusion index from the same time point as shown in B. Ten random 20× fields were imaged for myosin heavy chain and DNA, and number of cells with one nucleus or more than one nucleus was counted. This showed that 60 percent of control cells expressing myosin heavy chain had fused, while only 24 percent of Taxol treated and 40 percent of Nocodazole treated cells had fused. (TIF) [file pone.0031583.s001.tif]
